# Synergistic gut microbiome-mediated degradation of *Astragalus membranaceus* polysaccharides and *Codonopsis pilosula* polysaccharides into butyric acid: a metatranscriptomic analysis

**DOI:** 10.1128/spectrum.03039-24

**Published:** 2025-05-27

**Authors:** XinQian Rong, LingFeng Zhu, QingLong Shu

**Affiliations:** 1 College of traditional Chinese medicine, Jiangxi University of Chinese Medicine 74582, Nanchang, Jiangxi, China; Lerner Research Institute, Cleveland, Ohio, USA

**Keywords:** *Astragalus membranaceus *polysaccharide, *Codonopsis pilosula *polysaccharide, metatranscriptome, butyric acid, gut microbes

## Abstract

*Astragalus membranaceus* and *Codonopsis pilosula* are traditional Chinese medicines known for their tonifying effects, which are linked to the metabolism of their polysaccharide components in the gut. However, the role of gut microbiota in the degradation of these polysaccharides to butyric acid remains unclear. This study aims to investigate the *in vitro* degradation of polysaccharides from *Astragalus membranaceus* and *Codonopsis pilosula* by healthy mice fecal microbiota, focusing on butyric acid production and the associated microbial gene expression. We conducted an *in vitro* analysis of the degradation of homogeneous polysaccharides. from *Astragalus membranaceus* and *Codonopsis pilosula* using fecal microbiota cultures derived from healthy mice. The fecal microbiota was cultured with the polysaccharides for 48 hours, after which the degradation liquid was collected for butyric acid quantification and metatranscriptome analysis of the microbiota. The degradation of *Astragalus membranaceus* polysaccharide resulted in a significant increase in butyric acid levels compared to those produced from *Codonopsis pilosula* polysaccharide or fructooligosaccharide (control). Differential gene expression analysis indicated an upregulation of carbohydrate-active enzymes and genes associated with butyrate production during the degradation of *Astragalus membranaceus* polysaccharides. Additionally, the findings suggested that synergistic interactions between polysaccharide-degrading and butyrate-producing bacteria play a crucial role in the microbiota’s response to specific polysaccharides. This study highlights the potential of *Astragalus* polysaccharides to enhance butyric acid production through specific gut microbiota interactions, suggesting their beneficial effects on gut health and metabolism. Further research may provide insights into the therapeutic applications of these traditional medicines in modulating gut microbiota and improving health outcomes.

**IMPORTANCE:**

This study significantly advances our understanding of the role of gut microbiota in the metabolism of traditional Chinese medicinal polysaccharides, specifically those from *Astragalus membranaceus* and *Codonopsis pilosula*. By demonstrating that *Astragalus membranaceus* polysaccharide enhances butyric acid production more effectively than *Codonopsis pilosula* polysaccharide or fructooligosaccharides, the research highlights the potential of these natural compounds in modulating gut health. The identification of upregulated carbohydrate-active enzymes and butyrate production genes provides valuable insights into the microbial mechanisms underlying polysaccharide degradation. This work not only contributes to the field of microbiome research but also supports the development of functional foods and therapeutics aimed at enhancing gut health through targeted polysaccharide consumption.

## INTRODUCTION

In recent years, extensive research has been conducted on traditional Chinese medicine polysaccharides (1–3). The diversity of medicinal plant species in traditional Chinese medicine, as well as variations in the origin of the herbs, extraction methods, and purification techniques (4), has resulted in the purified polysaccharides exhibiting complex and diverse structural types. Medicinal herbs are among the most important sources of natural macromolecular polysaccharides (5). The pharmacological activities of different Chinese herbal polysaccharides vary, with the majority of studies focusing on their anti-inflammatory, antitumor, antioxidant, immunomodulatory, and prebiotic effects (6, 7). Among these, the prebiotic effects of polysaccharides are considered one of their most important properties. The prebiotic benefits of polysaccharides mainly stem from their ability to enrich beneficial microbial communities in the gut during their degradation by gut microbiota and the production of metabolites from this process (8). Moreover, multiple digestive simulation experiments (9, 10) and polysaccharide fluorescent labeling oral experiments (11) have indicated that high-molecular-weight polysaccharides are difficult to absorb and utilize in the small intestine and upper digestive tract, but once they enter the colon, they are degraded and utilized by the gut microbiota. Therefore, exploring the mechanism of interaction between polysaccharides and microbiota has become a key aspect of explaining the pharmacological activities of polysaccharides.

*Astragalus membranaceus* (Fisch.) Bunge and *Codonopsis pilosula* (Franch.) Nannf. are well-known medicinal and food-homologous plants (12, 13). In traditional Chinese medicine, *Astragalus membranaceus* and *Codonopsis pilosula* are used as classic “tonifying Qi herbs” and “tonifying Qi and blood herbs,” respectively, and the polysaccharides from these plants have received widespread research attention, demonstrating their therapeutic effects in models of colitis (14), immunosuppression (10), and inflammation (15). However, the precise mechanisms underlying the degradation of natural macromolecular polysaccharides by the gut microbiota are not yet clear.

Currently, research on polysaccharides and microbiota is mainly focused on *in vivo* experiments, emphasizing some of their pharmacological activities; however, studies on their underlying mechanisms are lacking. For mechanistic elucidation, *in vitro* experiments offer greater controllability (16). Therefore, the present study was conducted using an *in vitro* degradation model. Metatranscriptomics, unlike metagenomic sequencing, involves high-throughput sequencing of RNA transcripts from microbial communities, which provides information on microbial gene expression levels, identifies the functions of actively expressed genes, and, when used in longitudinal study designs, can monitor changes in microbial gene expression over time (17). Thus, this study used metatranscriptomics to determine microbial responses during the degradation of polysaccharides. Moreover, numerous studies have highlighted the beneficial effects of short-chain fatty acids on the body (18, 19), particularly butyric acid, which is known for its anti-obesity (20) and anti-inflammatory properties (21), and clinical trials have been successfully conducted. Acetate is produced via acetyl-CoA synthetase or the Wood-Ljungdahl pathway and serves as one of the potential precursors for butyrate synthesis (22). Consequently, butyric acid was selected as the final effector for measurement in this study. In addition, fructooligosaccharide (FOS) was chosen as a comparator (polysaccharide control group). FOS has demonstrated prebiotic effects *in vivo (*23) and is known to be degraded to butyric acid (24).

In this study, we examined the degradation of purified *Astragalus membranaceus* and *Codonopsis pilosula* polysaccharides *in vitro*, focusing on changes in the abundance of butyric acid-producing bacterial communities during degradation, differential expression of active genes, adjustments in metabolic pathways, and quantification of butyric acid. This study aimed to evaluate the metabolic responses of gut microbes during the fermentation and degradation of different polysaccharide substrates to butyric acid, with the goal of providing insights into the mechanisms of their polysaccharide-degrading activity.

## MATERIALS AND METHODS

### Materials

*Astragalus membranaceus* and *Codonopsis pilosula* were sourced from Jiangxi Jiangzhong Traditional Chinese Medicine Decoction Pieces Co., Ltd. and used in compliance with the 2020 edition of the Pharmacopoeia of the People’s Republic of China. Monosaccharide standards and dextran were obtained from Sigma-Aldrich (St. Louis, MO, USA). FOS (> 90% purity) was obtained from Beijing Solarbio Science and Technology Co., Ltd. (Beijing, China). Mouse feces were obtained from Hunan Slac Jingda Laboratory Animal Co., Ltd. (Hunan, China: SCXK(Xiang)2021-0002) from the same batch of healthy mice (8-week-old clean-grade ICR male mice). All other chemicals used in this study were of analytical grade and procured from Sigma Aldrich.

### Preparation of the fecal flora suspension

Fecal flora suspensions were prepared as previously described by Li et al. (25). Mouse feces (2 g) and 20 mL of phosphate buffer solution (pH 7.2) were agitated in a vortex mixer for 1 min. The impurities in the mouse feces were removed using a sterile gauze (placed in a beaker), and the supernatant containing the fecal bacterial suspension was collected. The suspension (16% vol/vol) was added to the preculture medium (5 g of tryptone, 2.5 g of yeast, 5 g of NaCl, 2.5 g of glucose, 3 g of maltose, and 500 mL of deionized water) and incubated at 37°C for 18 h under anaerobic conditions (25). The resulting pre-cultured mouse fecal flora suspension was designated as primal bacteria .

### Extraction and purification of polysaccharides

Polysaccharides from *Astragalus membranaceus* and *Codonopsis pilosula* were extracted using water extraction, followed by alcohol precipitation. The *Astragalus* and *Codonopsis pilosula* samples were soaked in water (1:6, wt/vol) for 20 min and subjected to a 30 min decoction. The resulting filtrate was collected, and the residue was subjected to another decoction in water (1:4, wt/vol) for 20 min. Subsequently, the two filtrates were combined, concentrated, and treated with Sevag reagent to remove the proteins. Macroporous resin (AB-8) was then used to remove the pigments. Membrane dialysis was used to remove small molecules and purify the polysaccharides.

The *Astragalus membranaceus* polysaccharide was subjected to chromatography on a DEAE-cellulose column (26 × 400 mm) and was sequentially eluted with distilled water at a flow rate of 4 mL/min, followed by elution with 0.1, 0.2, and 0.3 M NaCl, resulting in three fractions (E1, E2, and E3, respectively). The primary polysaccharide fraction (E1) was collected, concentrated, and dialyzed (MWCO 3,000 Da) against distilled water for 48–72 h. Subsequently, the polysaccharide solution was applied to a Sephacryl S-400 HR column (26 × 1,000 mm) and eluted with distilled water at a flow rate of 1.0 mL/min, resulting in two fractions (APS-E1F1 and APS-E1F2). The main component (APS-E1F1) was selected for further experiments and structural determination (26).

The *Codonopsis pilosula* polysaccharide was subjected to chromatography on a DEAE-cellulose column (26 × 400 mm) and was sequentially eluted with distilled water at a flow rate of 4 mL/min, followed by elution with 0.1, 0.2, and 0.3 M NaCl, resulting in two fractions (D1 and D2). The primary polysaccharide fraction (D1) was collected, concentrated, and dialyzed (MWCO 3,000 Da) against distilled water for 48–72 h. Subsequently, the polysaccharide solution was applied to a Sephacryl S-400 HR column (26 × 1,000 mm) and eluted with distilled water at a flow rate of 1.0 mL/min, resulting in one fraction (CPPS-D1N1) that was subjected to further experiments and structural determination (27).

### Structural characterization by NMR spectroscopy

The purified polysaccharide fractions (APS-E1F1 and CPPS-D1N1) were analyzed by 1D/2D NMR spectroscopy. Samples (~20 mg) were deuterium-exchanged by lyophilization with D₂O (99.9% D, Cambridge Isotope Laboratories) and dissolved in 0.6 mL D₂O. ¹H NMR (600 MHz) and ¹³C NMR (150 MHz) spectra were recorded on a Bruker Avance III HD spectrometer at 25°C. 2D spectra (HSQC, HMBC, and COSY) were acquired using standard pulse sequences. Chemical shifts were referenced to internal acetone (δH 2.225 ppm; δC 31.45 ppm). Glycosidic linkage patterns were determined through comparative analysis of anomeric proton/carbon resonances and cross-peaks in 2D spectra (26).

### Cultivation of the fecal flora suspension

The preparation and cultivation methods of the fecal slurry culture medium were modified from Martens et al. (28). Minimal medium contained 100 mM KH_2_PO_4_ (pH 7.2), 15 mM NaCl, 8.5 mM (NH_4_)_2_SO_4_, 4 mM L-cysteine, 1.9 µM hematin, and 200 µM L-histidine (prepared together as a 1,000 × solution), 100 µM MgCl_2_, 1.4 µM FeSO_4_·7H2O, 50 µM CaCl_2_, 1 µg mL^−1^ vitamin K3, and 5 ng mL^−1^ vitamin B12. Purified polysaccharides of APS, CPPS, and FOS were dissolved in distilled water to prepare 30% (wt/vol) polysaccharide solutions. The polysaccharide solutions were then combined with minimal medium in a volume ratio of 2:8 (vol/vol), sterilized (121°C, 0.1 MPa), and designated as the APS, CPPS, and FOS groups, respectively. The fecal slurry was added to each medium at a concentration of 5%. All cultures were incubated anaerobically in a TC-211B thermostatic bed (37°C, 160 rpm) for 48 h.

### Macrotranscriptome analysis of microbial communities

The cultures of APS, CPPS, and FOS groups were centrifuged at 30 × *g* for 10 min, followed by an additional centrifugation at 10,000 rpm for 10 min. The samples were obtained as degradation fluid and bacterial samples. Total RNA was extracted from the samples using TRIzol reagent (Invitrogen, US) according to the manufacturer’s instructions, and then ribosomal RNA was removed using a Ribo-Zero rRNA removal kit for bacteria (Illumina, US). The resulting residual RNA was quantified using a Thermo Scientific NanoDrop ND-2000 spectrophotometer (Thermo Scientific, Waltham, MA, USA), and RNA integrity was determined using an Agilent 2100 Bioanalyzer. The high-quality RNA samples (>10 µg, OD260/280 = 1.8-2.2, OD260/230 ≥ 2.0, RIN ≥ 6.5, 28S:18S ≥ 1.0) were used to construct a sequencing library. High-throughput RNA sequencing (RNA-Seq) was performed using TruSeq adapters and an Illumina HiSeq 2500 instrument (Shanghai Biozeron Biotechnology Co., Ltd., Shanghai, China). After the removal of host DNA and adapter contaminants, a set of high-quality reads was obtained, with an average of 6.74 gigabases of paired-end reads per sample. These samples were then subjected to further analysis (29).

#### Differential expression analysis

RNA-seq reads were aligned to the mouse gut microbiome reference genome (NCBI BioProject PRJNA385949) using HISAT2 (v2.2.1). Gene counts were quantified with featureCounts (v2.0.1) and normalized to TPM (transcripts per million). Differential expression between groups (APS/FOS and CPPS/FOS) was analyzed using DESeq2 (v1.30.1) with thresholds of log2 (fold change) >1 and adjusted *P*-value < 0.05 (Benjamini-Hochberg correction). KEGG pathway enrichment was performed using clusterProfiler (v4.0.5) with a significance cutoff of q-value <0.05.

#### Butyrate pathway prediction

The butyrate production potential was assessed by the following: (i) mapping differentially expressed genes (DEGs) to butyrate synthesis pathways (ko00650) in KEGG. (ii) Calculating pathway completeness scores based on the presence/expression of key enzymes (butyryl-CoA:acetate CoA-transferase, EC 2.8.3.8; phosphate butyryltransferase/butyrate kinase, EC 2.3.1.19 and EC 2.7.2.7).

### Determination of butyric acid

The degradation fluid was resuspended by adding 50 µL of 20% phosphoric acid, supplemented with 500 µM of 4-methylpentanoic acid as an internal standard. After centrifugation at 14,000 *g* for 20 min, the resulting supernatant was transferred into an injection vial. The samples were analyzed using GC–MS with an injection volume of 1 µL and a shunt ratio of 10:1. Separation of the samples was achieved using an Agilent DB-FFAP capillary column (30 m x 250 μm x 0.25 µm). The initial temperature was set at 90°C, followed by an increase at a rate of 10 °C/min until a temperature of 160°C is reached. Subsequently, the temperature was further increased at a rate of 40°C/min to 240°C and maintained for 5 min. Helium was used as the carrier gas at a flow rate of 1.0 mL/min. Quality control samples were periodically included in the sample queue to evaluate system stability and reproducibility. Mass spectrometry was performed using a 5977 B MSD mass spectrometer (Agilent Technologies) under specific conditions. These included an inlet temperature of 250°C, an ion source temperature of 230°C, a transfer line temperature of 250°C, and a quadrupole temperature maintained at 150°C. The mass spectrometry used an electron bombardment ionization source with an electron energy of 70 eV. Butyric acid levels in the samples were determined using the SCAN/SIM mode (27).

### Statistical analysis

Data are presented as the mean ± SD (*n* = 3) and analyzed for statistical differences using a one-way ANOVA followed by Duncan’s test. If *P* < 0.05, the differences were considered statistically significant. SPSS for Windows, Version 17.0 (SPSS Inc., Chicago, IL, USA), and GraphPad Prism software (version 7.0) were used for all the statistical analyses.

## RESULTS

### Polysaccharide structural analysis

APS-E1F1 and CPPS-D1N1 were identified as homogeneous polysaccharides. APS-E1F1 is composed of glucose, arabinose, galactose, and mannose in a molar ratio of 83.011:15.401:1.221:0.367, respectively, while CPPS-D1N1 consists of fructose and glucose in a molar ratio of 97.28:2.72. The molecular weights of APS-E1F1 and CPPS-D1N1 were 5.985 and 5.481 kDa, respectively. Methylation identification and nuclear magnetic resonance analysis revealed that APS-E1N1 is a polysaccharide with a backbone composed of →4)-α-D-Glcp-(1→ and →4, 6)-α-D-Glcp-(1→ units, with side chains of →6)-α-D-Glcp-(1→ and polymerized arabinose (26). CPPS-D1N1 is a polysaccharide with a main chain formed by connecting →1)-β-D-Fruf-(2→ units with a small amount of β-D-Fruf-(2→ . FOS are a group of carbohydrates, including disaccharides (GF2), trisaccharides (GF3), and tetrasaccharides (GF4), formed by sucrose and one to three fructose units linked by β−1,2-glycosidic bonds. Among them, *Astragalus membranaceus* polysaccharide had the most complex structure with the highest variety of glycosidic bonds, followed by *Codonopsis pilosula* polysaccharides, with FOS having the least complex structure.

### Polysaccharide degradation and carbohydrate-active enzymatic expression profile

**Fig 1:**
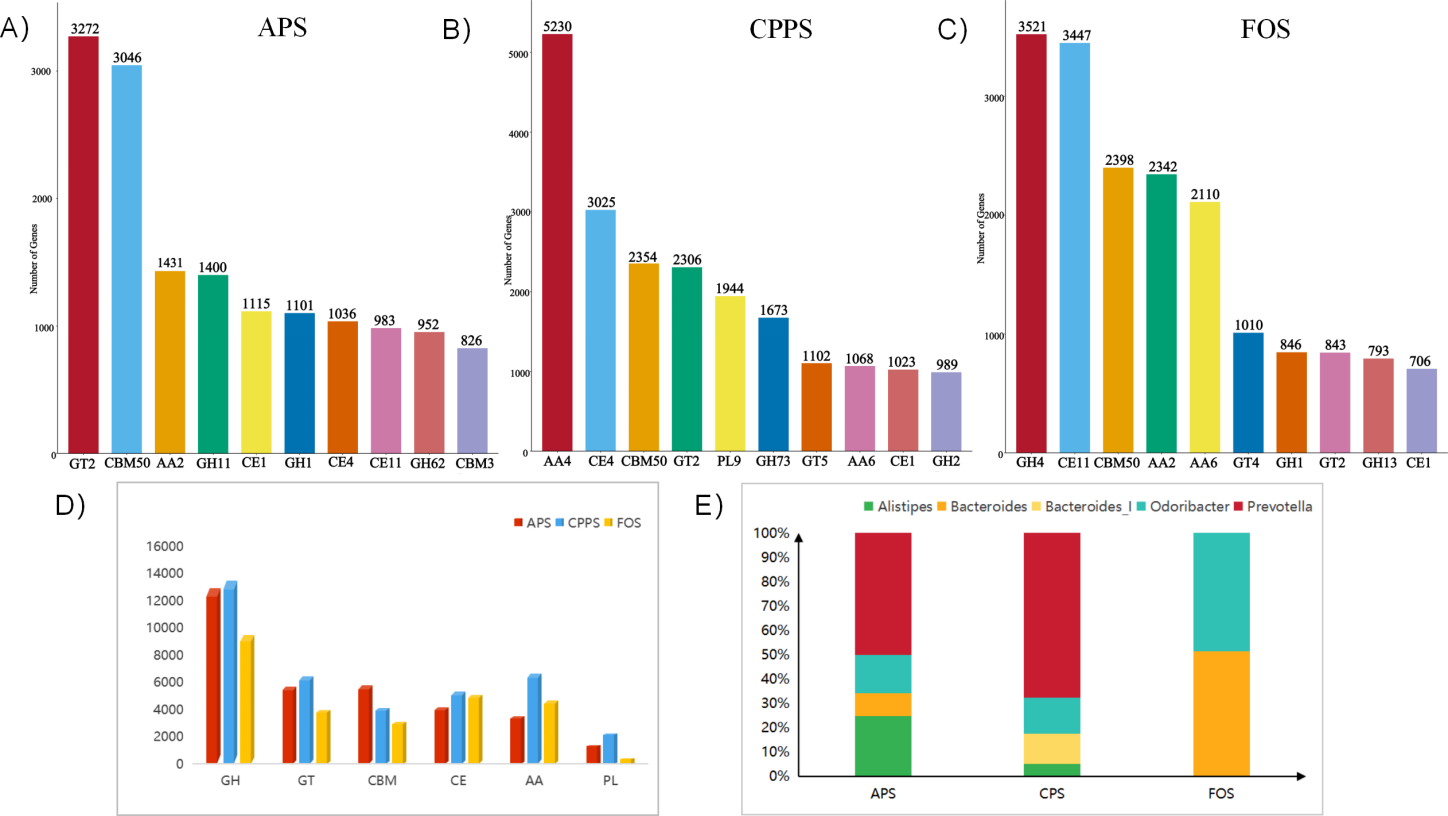
Expression profiles of carbohydrate-active enzymes (CAZymes) and Bacteroidetes enrichment: (A–C) Top 10 CAZymes expressed at the family level in the APS group (**A**), CPPS group (**B**), and FOS group (**C**). (**D**) Expression of CAZymes at the Class level across different groups. (**E**) Enrichment of Bacteroides in different groups. (GH: glycoside hydrolases, GT: glycosyl transferases, CBM: carbohydrate-binding modules, CE: carbohydrate esterases, AA: auxiliary activities, PL: polysaccharide lyases)

Carbohydrate-active enzymes (CAZymes) are classified into several families based on their catalytic mechanisms and substrate specificities. Glycoside hydrolases (GHs) are the most abundant CAZymes and specialize in cleaving glycosidic bonds in polysaccharides. They can function as independent catalytic modules or in complexes with carbohydrate-binding modules (CBMs) to enhance the efficiency in degrading complex substrates. Glycosyl transferases (GTs) catalyze the transfer of monosaccharide units to acceptor molecules, generating diverse oligosaccharide structures. Polysaccharide lyases (PLs) degrade acidic polysaccharides by cleaving glycosidic bonds via β-elimination, producing unsaturated hexenyluronic acid residues. Auxiliary activities (AAs), a class of oxidoreductases, assist in polysaccharide and lignin degradation, with AA1, AA2, and AA3 families being particularly prominent in Proteobacteria. Finally, carbohydrate esterases (CEs) hydrolyze ester-linked modifications (e.g., O- or N-acylations) on carbohydrates, facilitating further degradation by other CAZymes. Together, these enzyme families enable the gut microbiota to efficiently break down diverse dietary and host-derived glycans (30). The results revealed the relative abundances of GHs, GTs, CBMs, CEs, AAs, and PLs in descending order (Fig. 1D). The CAZymes expressed in the APS group belonged to 221 families, those in the CPPS group belonged to 211 families, and those in the FOS group belonged to 172 families. The top 10 CAZymes expressed in the APS group are shown in Fig. 1A, with GT2 exhibiting the highest expression, with a gene count of 3,272. In the CPPS group, the top 10 expressed CAZymes, as shown in Fig. 1B, were dominated by AA4, with a gene count of 5,230. In the FOS group, the top 10 expressed CAZymes, as depicted in Fig. 1C, were dominated by GH4 with a gene count of 3521. Members of the GH family primarily catalyze the hydrolysis of glycosidic bonds in diverse carbohydrate structures. In the APS group, the top expressed CAZymes include three GH families: GH11, GH1, and GH62, with GH11 targeting β(1-4) xylan glycosidic bonds, GH1 focusing on α/β cellulose glycosidic bonds, and GH62 acting on α-lactose glycosidic bonds. Conversely, in the CPPS group, the top expressed CAZymes include two GH families: GH73 and GH25, both capable of hydrolyzing β-cellulose glycosidic bonds. In the FOS group, the top expressed CAZymes belong to the GH4, GH11, and GH13 families, with GH4 possessing the ability to hydrolyze α/β cellulose glycosidic bonds and β-lactose bonds.

It is well known that Bacteroidetes are the primary gut microbiota encoding CAZymes in the human intestinal tract. Therefore, we further analyzed the gene enrichment patterns of Bacteroidetes across different carbon source substrates. As shown in Fig. 1E, five Bacteroides genera were enriched: *Alistipes*, *Bacteroide*s, *Bacteroides*_I, *Odoribacter*, and *Prevotella*. In the APS group, four genera were enriched (*Alistipes*, *Bacteroides*, *Odoribacter*, and *Prevotella*), with intergroup analysis showing relatively balanced proportions among these genera. The CPPS group also enriched four genera (*Alistipes*, *Bacteroides*_I, *Odoribacter*, and *Prevotella*), where Prevotella was identified as the dominant genus through intergroup analysis. In contrast, the FOS group only enriched two genera (*Bacteroides* and *Odoribacter*). These differential results likely stem from distinct degradation characteristics of the carbon substrates, particularly regarding CAZyme enrichment during polysaccharide degradation. Both APS and CPPS maintained a more balanced Bacteroidetes community structure, with APS demonstrating superior performance to CPPS—a finding that positively correlates with the structural complexity of different polysaccharides. In summary, our study demonstrates that APS, CPPS, and FOS as carbon sources can specifically regulate carbohydrate-active enzyme expression in gut microbiota. Different carbon sources led to significant differences in CAZyme expression profiles at both Class and family levels. This suggests that polysaccharide-degrading bacteria (primarily Bacteroidetes) possess the ability to adapt their enzymatic systems according to carbon source structure and properties, thereby achieving efficient utilization of diverse carbon sources.

### Quantitative analysis of butyric acid in degradation broths

**Fig 2:**
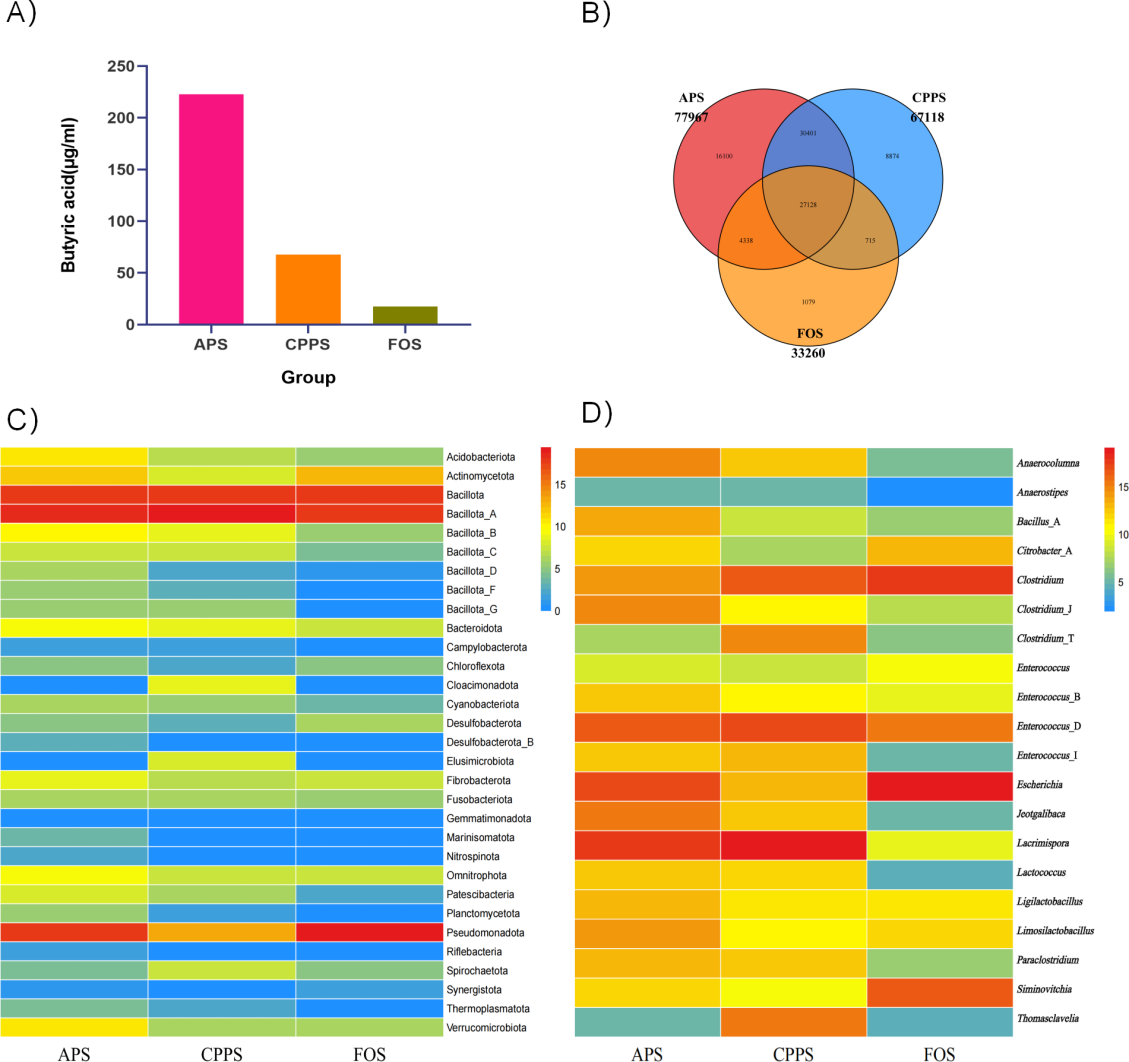
Statistics of differential genes in butyrate production across different substrates and expression profiles of functional microbial communities.(A) Butyric acid quantitative results. (B) Venn diagram of functional microbial gene expression across groups. (C) Phylum-level statistical heatmap of functional microbial differences. (D) Genus-level statistical heatmap of functional microbial differences .

Following the initial degradation of polysaccharides by microbial communities expressing CAZymes, subsequent processing is carried out by microbial communities possessing genes for butyric acid production, resulting in the generation of butyric acid as one of the final metabolic products of polysaccharide degradation. Therefore, we initially quantitatively determined the butyric acid content in the fermentation broths, with results shown in Fig. 2A. Interestingly, despite using an equal dosage of polysaccharides and utilizing microbial communities from identical samples for each experiment, the production of butyric acid varied significantly. The butyric acid content in the degradation broth of *Astragalus membranaceus* polysaccharides far exceeded that of *Codonopsis pilosula* polysaccharides and FOS, with the former slightly surpassing the latter. This significant difference is likely attributable to differential gene expression in the butyric acid production pathway. Butyrate is produced from carbohydrates via glycolysis by combining two molecules of acetyl-CoA to form acetoacetyl-CoA, following a sequential reduction to butyryl-CoA. Two pathways are known for the final step of butyrate formation: butyryl-CoA:acetate CoA transferase and butyrate kinase. Therefore, we further characterized the enrichment of the microbial genes involved in butyric acid production.

### Butyric acid metabolic pathway and related enzymatic expression profile

**Fig 3:**
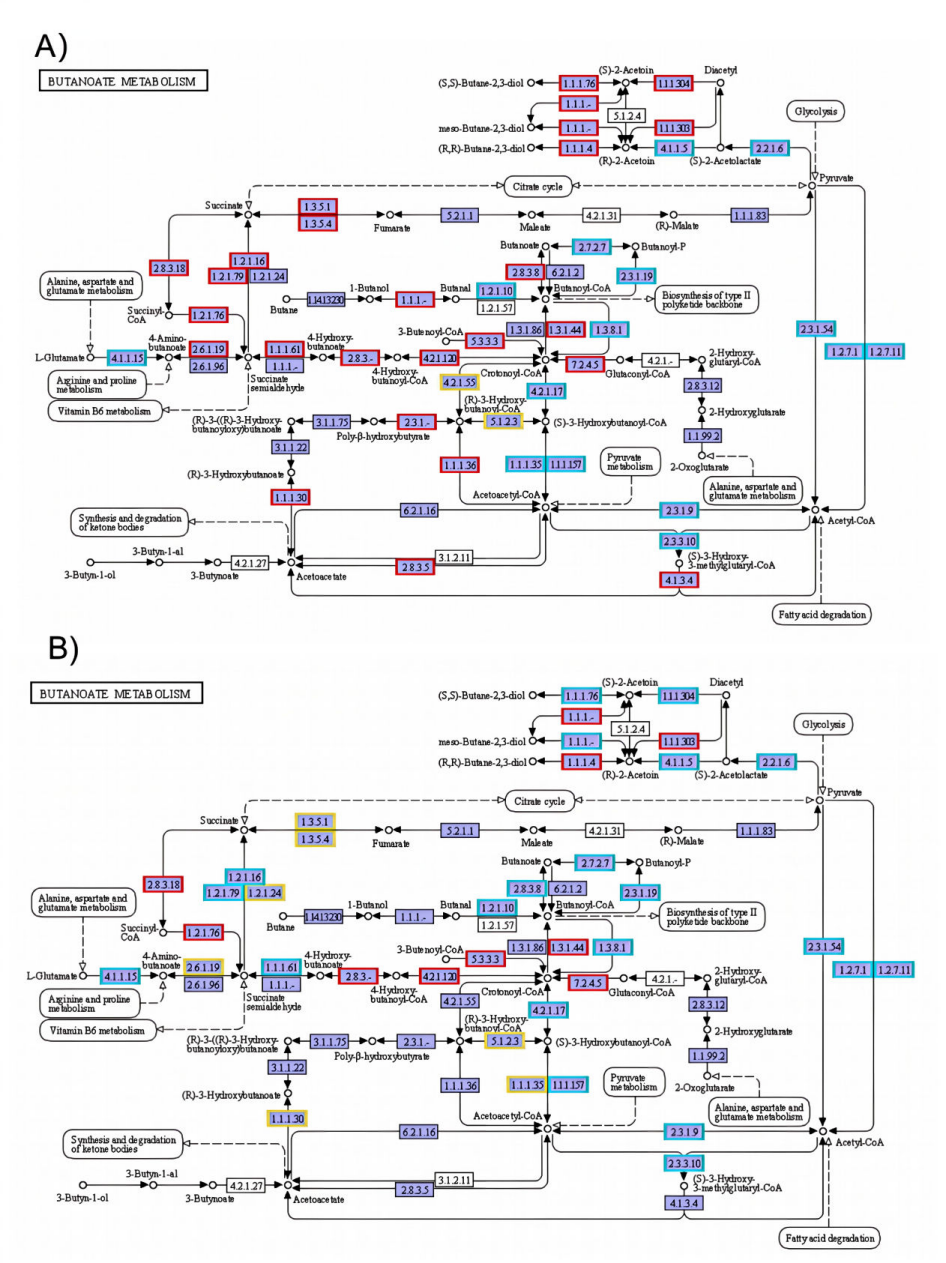
Bacterial enzymes involved in butanoate metabolism and changes in their expression in the presence of polysaccharides from *Astragalus* and *Codonopsis pilosula*. Differentially regulated enzymes in the APS (**A**) and CPPS (**B**) groups using enzymatic expression in the FOS group as the baseline. All gene products with blue-filled boxes represent background genes present in the species tested in this transcriptome, while gene products with white-filled boxes indicate genes not belonging to the species tested in this transcriptome. Genes with red, yellow, or sky blue borders indicate differentially expressed genes annotated to this metabolic pathway, representing upregulated, downregulated, and both upregulated and downregulated genes, respectively. The number in each box corresponds to the Enzyme Commission number identifying the enzyme.

A total of 451 genes related to the butyric acid metabolic pathway were detected across the three samples: 329 in the APS group, 76 in the CPPS group, and 46 in the FOS group. To compare differential gene enrichment, we used the FOS group as the standard for the butyric acid metabolic pathway. Compared with the FOS group, the APS group exhibited 287 upregulated and 49 downregulated genes, demonstrating a statistically significant difference in butyric acid-related gene expression between the two groups (*P* = 0.001; Fig. 3A). In contrast, the CPPS group showed 135 upregulated and 83 downregulated genes relative to the FOS group, with no statistically significant difference in butyric acid-related gene expression (*P* = 0.363; Fig. 3B). Further analysis was conducted on the enzymatic expression profiles during metabolic processes. A total of 56 enzymes involved in the butyric acid metabolic pathway were included in the transcriptome analysis. Compared to the FOS group, the APS group had 26 upregulated and 2 downregulated enzymes, while the CPPS group had 10 upregulated enzymes and 7 downregulated enzymes.

We further compared the expression differences of three key enzymes involved in butyrate synthesis pathways among three polysaccharide treatment groups (APS, CPPS, and FOS) (Table 1). The results demonstrated that the APS group exhibited significantly higher expression levels of all key enzymes (but, ptb, and buk) compared to CPPS and FOS groups, indicating its potentially superior efficiency in promoting gut microbiota-derived butyrate production. The CPPS group showed intermediate expression levels, although with slightly higher butyryl-CoA transferase (but) expression than the APS group, suggesting its specific advantage in the CoA-transferase pathway. The FOS group displayed the lowest enzymatic expression, consistent with its nature as a simple carbon source, resulting in relatively weaker butyrate production capacity.

**TABLE 1:** Expression levels of key enzymes in butyrate production pathways across different polysaccharide groups

Key enzyme	Corresponding pathway	Enzyme number	Gene	APS	CPPS	FOS
Butyryl-CoA:acetate CoA-transferase	CoA-transferase pathway	[EC:2.8.3.8]	but	3	4	1
Phosphate butyryltransferase	PTB/Buk pathway	[EC:2.3.1.19]	ptb	9	4	3
Butyrate kinase	PTB/Buk pathway	[EC:2.7.2.7]	buk	17	7	5

**Fig 4:**
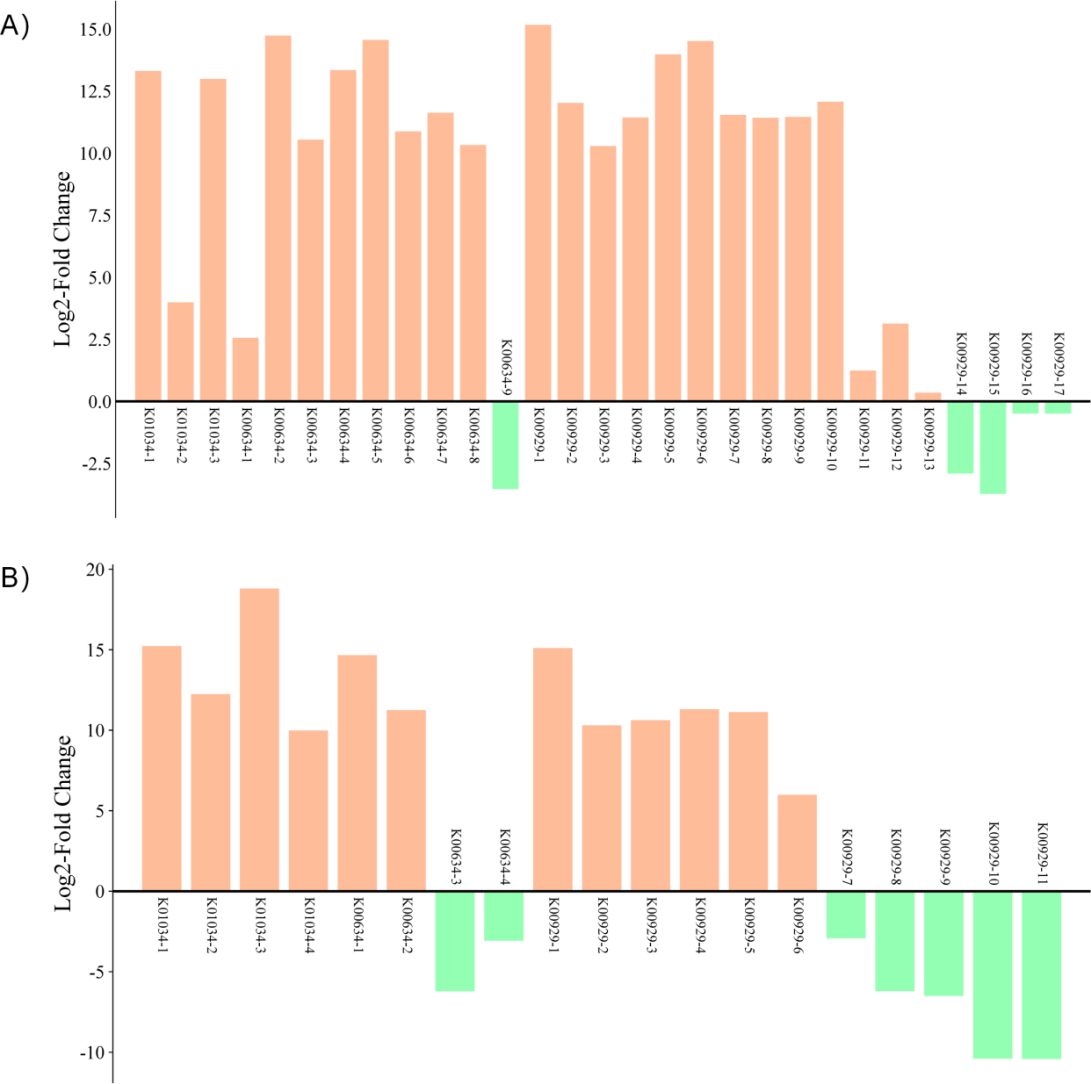
Enrichment of expression differences in three key butyrate-producing genes APS vs FOS. (**A**) CPPS vs FOS (**B**). The KEGG database IDs for these three key genes are as follows: K01034, K00634, and K00929.

Notably, the PTB/Buk pathway (ptb +buk) was highly active in the APS group, likely serving as the primary driver for its high butyrate yield. Fig. 4A and B illustrates the expression differences (Log2-Fold Change) of three key butyrate synthesis genes (KEGG IDs: K01034, K00634, and K00929) between APS/FOS and CPPS/FOS groups, fully aligning with the enzymatic expression trends in Table 1. These findings collectively demonstrate that polysaccharide complexity (APS >CPPS > FOS) positively correlates with the activation level of microbial butyrate synthesis pathways, supporting the hypothesis that “carbon source structure drives metabolic specialization in gut microbiota.” The APS group achieves high butyrate production through dual-pathway synergy (PTB/Buk-dominant + CoA-transferase-assisted), which may render it particularly suitable for gut inflammation modulation, given butyrate’s well-established anti-inflammatory properties.

### Construction of functional microbial enrichment based on gene expressions of functional genes

The gene set was aligned against the Non-Redundant Database (NR) using BLASTP, and species annotation was obtained through the corresponding taxonomic information database of NR. The abundance of each species was then calculated by summing the abundances of its corresponding genes. As shown in Fig. 2B, the APS, CPPS, and FOS groups exhibited 77,967, 67,118, and 33,260 gene expression profiles, respectively, with the functional microbial gene abundance ranking as the APS group >CPPS group >FOS group.

Next, based on the enriched gut bacterial gene expression, we statistically analyzed the species abundance at the phylum and genus taxonomic levels to construct differential abundance profiles at the phylum (Fig. 2C) and genus (Fig. 2D) levels. At the phylum level, Bacillota and its subgroup A were the most dominant enriched phyla across all three groups. In the APS group, Bacillota subgroups C, D, F, and G also exhibited a relatively high abundance. Notably, Bacillota are recognized as core butyrate producers in the gut. The abundance ranking of Bacillota across groups was APS group >CPPS group >FOS group, which positively correlated with the butyrate quantification results obtained in Polysaccharide degradation and carbohydrate-active enzymatic expression profile of this article.

At the genus level, we analyzed the expression profiles of the top 20 enriched genera. Among these, *Anaerocolumna*, *Anaerostipes*, *Lacrimispora*, *Thomasclavelia*, *Paraclostridium*, and *Clostridium* (including subgroups J and T) were identified as classical butyrate-producing genera. In the APS and CPPS groups, the most dominant enriched genus was *Lacrimispora*, a core butyrate producer. The FOS group also showed a relatively high expression of *Lacrimispora*, indicating its high adaptability to different carbon substrates. Additionally, in the APS group, *Anaerocolumna*, *Paraclostridium*, and *Clostridium* (including subgroup J) were present at high abundance levels, suggesting their major role in butyrate production from APS. In the CPPS group, *Anaerocolumna*, *Paraclostridium*, *Thomasclavelia*, and *Clostridium* (including subgroups J and T) exhibited high abundance, with *Thomasclavelia* being significantly more abundant than in other groups, highlighting its key role in butyrate production from CPPS. In the FOS group, *Lacrimispora* and *Clostridium* were highly enriched, indicating their primary contribution to butyrate production from FOS.

Distinct carbon source groups exhibited substantial unique gene expression profiles, demonstrating that gut microbiota can specifically regulate gene expression in response to different carbon sources to facilitate their utilization and metabolism. These unique genes may participate in pathways related to specific carbon source metabolism, enzyme synthesis, and microbe-substrate interactions, reflecting the adaptability and metabolic flexibility of the microbiota toward carbon sources.

## DISCUSSION

Butyric acid is a crucial short-chain fatty acid in the human gut, with its production and metabolism linked to the treatment of various diseases, including obesity, diabetes, inflammatory bowel diseases, colorectal cancer, and neurological disorders (31). Its mechanisms of action include serving as an energy source for the intestinal barrier and microbiota, as well as functioning as an immunomodulatory bioactive molecule (32). Notably, immune activation—a well-documented pharmacological effect of traditional Chinese medicine polysaccharides[33]—often coincides with elevated gut butyrate levels (34). This phenomenon is strongly associated with the structural specificity of digestible polysaccharides (35). However, the mechanistic connections between polysaccharide structure, microbial degradation, and butyric acid production remain poorly understood. We identified three primary challenges in the study of polysaccharides, microbiota, and butyric acid: first, the complexity of natural macromolecular polysaccharides; second, the intricate and hierarchical participation of microbiota in polysaccharide degradative metabolism; and third, the complexity of the factors associated with butyric acid production efficiency.

Current polysaccharide structural analyses (e.g., methylation and NMR) face limitations in characterizing low abundance or weakly signaling structures (36). In our study, APS-E1F1 and CPPS-D1N1 exhibited minor unresolved linkages (e.g., 4 Gal(p) in APS; α-D-Glcp-(1→ in CPPS) due to signal attenuation. To address this, we employed metatranscriptomics to profile CAZymes involved in polysaccharide degradation (37). The CAZyme abundance hierarchy (APS >CPPS > FOS) mirrored polysaccharide structural complexity, with APS recruiting the most diverse CAZymes (221 families), including GH11 (targeting β−1,4-xylan) and GH1 (cleaving α/β-cellulose), while FOS primarily activated GH4 (α/β-cellulose hydrolysis) (Fig. 1A–C). This suggests CAZyme expression profiles may serve as a proxy for inferring polysaccharide structural features.

Polysaccharide degradation involves a microbial cascade: CAZyme-producing bacteria (e.g., *Alistipes*, *Bacteroide*s, *Bacteroides*_I, *Odoribacter*, and *Prevotella*) initiate breakdown, followed by butyrogenic taxa (e.g., *Anaerocolumna*, *Anaerostipes*, *Lacrimispora*, *Thomasclavelia*, *Paraclostridium*, and *Clostridium*) metabolizing oligosaccharides into butyrate (38). Despite equal polysaccharide doses, APS yielded significantly higher butyrate than CPPS and FOS (Fig. 2A), correlating with gene upregulation: APS upregulated 287 butyrate-pathway genes (vs. FOS; *P* = 0.001), including but (CoA-transferase) and ptb/buk (PTB/Buk pathway) (Table 1; Figure 4A). Microbial specialization: APS enriched a balanced Bacteroidetes consortium (*Alistipes* and *Bacteroides*), while CPPS favored *Prevotella* (Fig. 1E). Bacillota (e.g., *Anaerocolumna* and *Paraclostridium*) dominated butyrate production in APS, whereas CPPS relied on *Thomasclavelia* (Fig. 2D).

The superior butyrogenic efficiency of APS may arise from (i) structural diversity: APS’s heterogeneous backbone (→4)-α-D-Glcp-(1→ with →6)-α-D-Glcp-(1→ side chains) and high monosaccharide diversity (glucose:arabinose:galactose = 83:15:1) provided broader substrate specificity for CAZymes. (ii) APS activated dual butyrate pathways (PTB/Buk-dominated + CoA-transferase-assisted), while CPPS relied more on CoA-transferase (Fig. 4B). (iii) APS-degrading B. subtilis and butyrogenic Lacrimispora exhibited cooperative metabolism, whereas CPPS-associated *P. simplicissimum* and *Thomasclavelia* showed less efficient coupling.

Our findings highlight a structure-function continuum. Polysaccharide complexity (APS > CPPS > FOS) drives CAZyme recruitment and butyrate-pathway activation, mediated by microbial consortia (Bacteroidetes degradation → Bacillota fermentation) (39, 40). APS’s high butyrate yield (via PTB/Buk) underscores its promise for gut inflammation modulation (41). Future studies should explore how polysaccharide structures (e.g., in APS/CPPS) influence microbial degradation kinetics. While substrate utilization involves both competitive (e.g., *Prevotella dominance* in CPPS) and cooperative interactions (e.g., Bacillus-*Lacrimispora* in APS), gut microbiota homeostasis likely emerges from their dynamic balance.

This work establishes a clear structure-function continuum where polysaccharide complexity determines microbial degradation strategies and butyrate production efficiency. The APS-microbiota-butyrate axis represents a promising target for therapeutic development, while our analytical framework provides a blueprint for studying other polysaccharide-microbiota interactions.

## Data Availability

Sequencing data have been uploaded to the official NCBI website. Please reference PRJNA1261438
